# First detection of porcine circovirus 4 (PCV-4) in Europe

**DOI:** 10.1186/s12985-023-02181-1

**Published:** 2023-10-10

**Authors:** Rocío Holgado-Martín, José Luís Arnal, Marina Sibila, Giovanni Franzo, Desireé Martín-Jurado, David Risco, Joaquim Segalés, Luís Gómez

**Affiliations:** 1 Departamento de Medicina Animal, Unidad de Anatomía Patológica y Anatomía Comparada, Facultad de Veterinaria de Cáceres, Universidad de Extremadura, Cáceres, 10003 Spain; 2 Exopol, Autovaccines and Veterinary Diagnostics, Polígono Río Gállego D/14, San Mateo de Gállego, Zaragoza, 50840 Spain; 3 Programa de Sanitat Animal, IRTA, Centre de Recerca en Sanitat Animal (CReSA), Universitat Autònoma de Barcelona (UAB), Campus, Bellaterra, 08193 Spain; 4 Unitat Mixta d’Investigació IRTA-UAB en Sanitat Animal, Centre de Recerca en Sanitat Animal (CReSA), Campus de la Universitat Autónoma de Barcelona (UAB), Bellaterra, 08193 Spain; 5 WOAH collaborating Centre for the Research and Control of Emerging and Re-Emerging Swine Diseases in Europe (IRTA-CReSA), Bellaterra, 08193 Spain; 6 Department of Animal Medicine, Production and Health (MAPS), Padua University, Legnaro, 35020 Italy; 7 Departament de Sanitat i Anatomia Animals, Facultat de Veterinària, Universitat Autònoma de Barcelona, Bellaterra, 08193 Spain

**Keywords:** Porcine circovirus 4 (PCV-4), Wild boar, Iberian pig, Spain, Italy

## Abstract

Porcine circovirus 4 (PCV-4) is a novel virus recently discovered (2019) in domestic pigs from China, although several studies have proven its circulation since 2008. Later, PCV-4 was also detected in wild boar populations from China and domestic pigs from South Korea and Thailand. Currently, Asia is so far the only continent where this novel virus has been reported; few studies carried out in South America and Europe failed in the attempt to detect it. The objective of this Comment is to communicate the first detection of PCV-4 in Europe, specifically in wild boar and domestic pigs from Mid-South-Western Spain. A retrospective study was carried out on wild boar and domestic pigs, both extensively (Iberian breed) and intensively raised, from Spain and Italy, sampled between 1998 and 2022. PCV-4 genome detection was attempted using different conventional or quantitative real time PCR (qPCR) protocols and some positive results were confirmed through Sanger sequencing. A total of 57 out of 166 (34.3%) Spanish wild boar and 9 out of 223 (4%) Iberian pigs (both geographically located in the Mid-South-Western Spain) were qPCR positive, while the rest of tested animals from North-Eastern Spain and Italy were negative. Partial sequences of Rep or Cap genes of selected samples confirmed the presence of PCV-4. The relatively high prevalence in wild boar and the low one in Iberian pigs from the same areas suggests intra- and interspecific transmission, being the wild boar a potential viral reservoir. The epidemiological and clinical importance of these findings are currently unknown, but guarantees further research on this novel virus.

## Background

Porcine circovirus 4 (PCV-4) is a novel virus from the *Circoviridae* family, which comprises small icosahedral and non-enveloped viruses with single-stranded and circular DNA genome. It was discovered in 2019 in domestic pigs (*Sus scrofa domestica*) in China [1]. However, retrospective studies have indicated that infection could be traced back to 2008 at least [2, 3]. Subsequently, it has also been found in wild boar (*Sus scrofa*) in China [4]. In addition, PCV-4 DNA has been detected in other domestic species such as dairy cows and dogs in China [5, 6]. Outside China, PCV-4 genome has been found only in Korea and Thailand [7, 8], although several surveys have been conducted in other continents, such as South America and Europe [9, 10]. Therefore, Asia is the only continent where the virus has been detected so far. The limited sequences available point out the existence of two different genotypes of PCV-4 (PCV-4a and PCV-4b). Both genotypes have been found in China, although only the PCV-4b one has been identified in Thailand and South Korea [7, 8].

PCV-4 DNA has been detected in domestic pigs affected by respiratory disorders and porcine dermatitis and nephropathy syndrome (PDNS), as well as in apparently healthy animals [1]. Detection of PCV-4 genome in wild boar has not been associated to any specific clinical condition [4]. Interestingly, PCV-4 has been detected in co-infection with other porcine circoviruses [4].

Most of the studies on PCV-4 have been based on conventional or real time quantitative PCR (qPCR) methods; only one used an in situ hybridization technique [8], although the obtained results were not conclusive.

## Main text

The incidence of pig infectious emerging and re-emerging diseases is continuously increasing [11]. These infections tend to spread easily because of the globalization of trade and the movement of people and animals worldwide [12]. In Spain, the porcine industry is mainly based on intensive farms; however, the Iberian pig production is still featured by a semi-extensive system specially in Mid-South-Western Spain, where pigs usually have contact with wild animals, such as wild boar [13]. Taking into account the economic and social importance of the swine industry in Spain as well as in Europe, the spread of emerging pathogens such as PCV-4 should be considered a matter of concern. Therefore, the objective of this Comment is to communicate the presence of PCV-4 in Europe, at least in Spain.

Table 1 summarizes the results of a collaborative and retrospective study carried out by several research groups from Spain and Italy using wild boar and domestic pig samples taken in different time periods between 1998 and 2023. Analyzed samples were collected from previously existing collections from epidemiological studies (in the case of wild boar), diagnostic cases or monitoring sampling.

**Table 1:** Wild boar and domestic pig samples tested by qPCR/PCR methods for the detection of PCV-4 in Spain and Italy. Percentage of qPCR/PCR positive ones are given attending to the type of sample and/or clinical condition studied

Country	Time period	Suidae species tested	Tested sample or clinical condition	Number of studied samples	Number of positive samples (%)
Spain	2011–2015	Wild boar*	Lymph nodes	166	56 (33.7%)
			Serum^1^	90	8 (8.9%)
	2021–2023	Iberian pig*	Digestive^2^	99	7 (7%)
			Lung	57	2 (3.5%)
			Reproductive^3^	14	0 (0%)
			Others^4^	53	0 (0%)
	2018–2021	Commercial breed pig**	Digestive^2^	73	0 (0%)
			Lung	32	0 (0%)
			Reproductive^2^	66	0 (0%)
			Others^4^	13	0 (0%)
	1998–2020	Commercial breed pig**	PDNS cases^5^	100	0 (0%)
	2018–2020	Commercial breed pig**	Serum^6^	900	0 (0%)
Italy	2014–2015	Wild boar	Serum	187	0 (0%)
	2017–2018	Wild boar	Serum	29	0 (0%)
	2021–2022	Wild boar	Lung and lymph node homogenate	103	0 (0%)
		Commercial breed pig	Serum	45	0 (0%)
		Backyard pigs	Lung and lymph node homogenate	68	0 (0%)

*Samples from animals located in Mid-South-Western Spain; **Samples from animals located in North-Eastern Spain

^1^The 90 sera from Spanish wild boar belonged to the 166 ones from which lymph nodes were tested

^2^Digestive samples included intestines, feces and rectal swabs

^3^Reproductive samples included foetus homogenates, placenta and fetus stomach content

^4^Others included sera and oral fluids from monitoring analytics, joint swabs from poliarthritis illness and organs such as liver, spleen or brain from systemic disorders and sudden deaths

^5^Mixture of tissues (lymph nodes, tonsil, spleen and lung) of porcine dermatitis and nephropathy syndrome (PDNS) affected pigs

^6^Serum of 3 day-old healthy piglets

Different PCR methodologies were used for the purpose, including techniques already published in the literature [1, 9] and new in-house conventional and real time PCR protocols targeting the Rep gene. Sanger sequencing was carried out on Rep or Cap gene from those samples whose Cq value was lower than 32, to maximize the likelihood of obtaining good-quality sequences [14]. The obtained nucleotide sequences were analysed and assembled using MEGA11 Molecular Evolutionary Genetics Analysis version 11 software [15].

A total of 57 different Spanish wild boar were positive for PCV-4 qPCR; specifically, 56 lymph nodes and 8 sera, with Cq values ranging 21–38 and 27–36, respectively. All positive serum samples were also positive to lymph node, except one. The Rep gene of 6 strains detected from positive lymph nodes was partially sequenced (GenBank accession numbers OR367318-OR367323) confirming the detection of PCV-4 genome. On the other hand, a total of 9 samples (seven intestines and two lungs) from different Iberian pig ranches were qPCR positive (Cq ranging from 31 to 37). The Cap gene from three samples was partially sequenced, confirming as well the detection of PCV-4 genome (GenBank accession numbers OR333699, OR333700 and OR359763). All qPCR positive animals were geographically located in Mid-South-Western Spain. Wild boar PCV-4 sequences were genetically identical to each other in the considered Rep region. A close relationship (identity > 99.7) was detected with several other Chinese and South Korean strains. The only exception was represented by strain OR367323, which showed a p-distance = 0.02 (i.e. 98% genetic identity) compared to the other Spanish wild boar sequences and a p-distance of 0.015 with a Chinese strain (OP497960) collected in 2022. A similar distance (p-distance = 0.017) was detected with other Chinese strains sampled since 2017 (i.e. MT882411, MK986820, NC_055580). The Cap sequences obtained from the Iberian pigs had the closest relationship with strains collected from China and South Korea since 2017 (p-distance = 0.007). PCV-4 DNA was not detected in the other commercial pig samples from Spain (North-Eastern Spain) or in domestic swine (backyard and intensively raised) and wild boar from Italy (Table 1).

## Conclusions

**Fig. 1:**
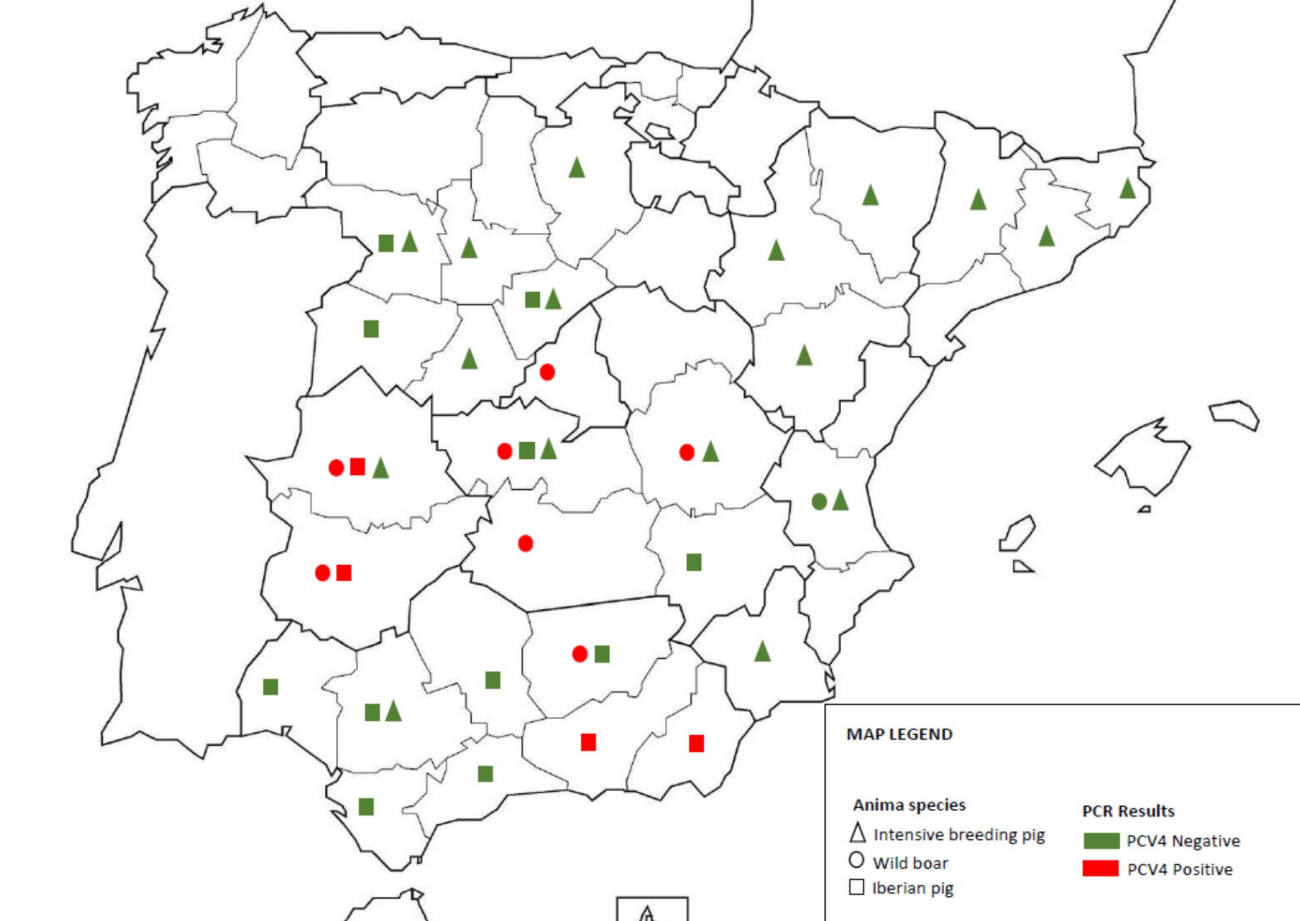
Map of Spain showing the location (provinces) of PCV-4 PCR positive wild boar (red circles) and Iberian pigs (red squares). The green circles, squares and triangles show the provinces in which wild boar, Iberian pigs and intensive breeding pigs, respectively, yielded negative PCV-4 PCR negative results

To the best of our knowledge, the detection of PCV-4 in wild boar and Iberian pigs represents the first report of this virus in Europe. Interestingly, most of infected animals were wild boar, which may suggest their potential reservoir role. Moreover, analysed animals were located in the Mid-South-Western Spain (Fig. 1), which is the traditional area of Iberian pig rearing in outdoor conditions. Since the Iberian pig semi-extensive breeding system is a special ecosystem called “Dehesa”, where domestic animals and wild fauna can interact directly or indirectly, it is tempting to speculate the potential interspecific transmission of pathogens from wild boar to Iberian pig [13, 16]. The lack of detection of PCV-4 infection in intensively reared pigs could be related to the higher biosecurity level in this type of farms and/or the lack of effective contact with the wild. In any case, the route of entry of PCV-4 in wild boar and Iberian pigs in Spain remains unknown.

A recent study carried out in Spain and Italy yielded negative results in the attempt to detect PCV-4 in serum and tissue homogenates collected from both wild boar and domestic pigs [9]. One possible explanation is that these previous surveys mainly included samples belonging to intensive breed pigs from Italy and North-Eastern Spain. Also, the previously tested wild boar were only from Italy, and novel results confirmed again negativity for PCV-4 in these populations. In addition, tested Italian backyard pigs were negative for the new virus. In consequence, PCV-4 seems to be still absent in pigs from these geographic areas.

In summary, this Comment confirms that PCV-4 has been retrospectively detected in Europe, specifically in wild boar and Iberian pigs reared in Mid-South-Western Spain. Interestingly, no evidence of infection has been found in domestic swine from North-Eastern Spain or in pigs and wild boar from Italy. If this epidemiological scenario means a limited geographical distribution of PCV-4 in Spain or in Europe requires further investigations. No clue on the potential pathogenicity of PCV-4 in domestic swine or wild boar can be drawn from the current exploratory study.
